# Ultrashort Self-Assembling Peptide Hydrogel for the Treatment of Fungal Infections

**DOI:** 10.3390/gels4020048

**Published:** 2018-05-22

**Authors:** Alyaa A. Albadr, Sophie M. Coulter, Simon L. Porter, Raghu Raj Singh Thakur, Garry Laverty

**Affiliations:** 1 Biofunctional Nanomaterials Group, School of Pharmacy, Queen’s University Belfast, Medical Biology Centre, 97 Lisburn Road, BT9 7BL Belfast, Northern Ireland; aalbadr01@qub.ac.uk (A.A.A.); scoulter14@qub.ac.uk (S.M.C.); sporter13@qub.ac.uk (S.L.P.); r.thakur@qub.ac.uk (R.R.S.T.); 2 Biology Department, Faculty of Science, Basra University, Basra, Iraq

**Keywords:** peptide, self-assembly, nanomaterial, hydrogel, aspergillosis, candidiasis

## Abstract

The threat of antimicrobial resistance to society is compounded by a relative lack of new clinically effective licensed therapies reaching patients over the past three decades. This has been particularly problematic within antifungal drug development, leading to a rise in fungal infection rates and associated mortality. This paper highlights the potential of an ultrashort peptide, (naphthalene-2-ly)-acetyl-diphenylalanine-dilysine-OH (NapFFKK-OH), encompassing hydrogel-forming and antifungal properties within a single peptide motif, thus overcoming formulation (e.g., solubility, drug loading) issues associated with many currently employed highly hydrophobic antifungals. A range of fungal susceptibility (colony counts) and cell cytotoxicity (MTS cell viability, LIVE/DEAD staining^®^ with fluorescent microscopy, haemolysis) assays were employed. Scanning electron microscopy confirmed the nanofibrous architecture of our self-assembling peptide, existing as a hydrogel at concentrations of 1% *w*/*v* and above. Broad-spectrum activity was demonstrated against a range of fungi clinically relevant to infection (*Aspergillus niger*, *Candida glabrata*, *Candida albicans*, *Candida parapsilosis* and *Candida dubliniensis*) with greater than 4 log_10_ CFU/mL reduction at concentrations of 0.5% *w*/*v* and above. We hypothesise antifungal activity is due to targeting of anionic components present within fungal cell membranes resulting in membrane disruption and cell lysis. NapFFKK-OH demonstrated reduced toxicity against mammalian cells (NCTC 929, ARPE-19) suggesting increased selectivity for fungal cells. However, further studies relating to safety for systemic administration is required, given the challenges toxicity has presented in the wider context of antimicrobial peptide drug development. Overall this study highlights the promise of NapFFKK-OH hydrogels, particularly as a topical formulation for the treatment of fungal infections relating to the skin and eyes, or as a hydrogel coating for the prevention of biomaterial related infection*.*

## 1. Introduction

Antimicrobial resistance is a severe and increasing threat to society. Overuse of and bacterial resistance to antibiotics receives the majority of public attention; however, the emergence of fungi resistant to multiple therapies has led to an urgent need for new, effective and safe antifungal agents [1]. The incidences of fungal infection are increasing due to factors such as an ageing population, increasing the prevalence of underlying disease (AIDS, cancer, diabetes) [2]. Modern medicine has also contributed to an increase in fungal infections directly through the use of medical devices (e.g., catheters), immunosuppressive therapies and antibiotics [3,4]. Candidiasis and aspergillosis are the two most common forms of fungal disease [5]. *Candida* is a yeast that exists primarily within the gastrointestinal tract as a commensal microorganism. However, if the normal microflora is disturbed, for example due to use of antibiotics, it becomes an opportunistic pathogen [6]. In the United Sates *Candida* species are the fourth most common pathogen implicated in septicaemia and represent a major source of nosocomial infections [7,8]. *Aspergillus* is a saprophytic filamentous mould, widely distributed throughout the natural environment, commonly found in areas of damp and on decaying vegetation. Their spores are widespread within air but only tend to cause infection in patients with lowered immunity or damaged lungs. *Aspergillus niger* is also an opportunistic pathogen, leading to serious infections such as pulmonary aspergillosis [9]. The severity of fungal septicaemia and the importance of prevention or early treatment are highlighted by severe mortality rates. Between 30–50% episodes of invasive aspergillosis and 50% of candidiasis result in death. Collectively, the two species are responsible for more than 75% of all mortalities related to fungal infections [10,11].

Four major classes of antifungal drugs currently exist for the treatment of systemic fungal infections: polyenes, pyrimidine analogues, echinocandins and triazoles [12,13]. Alternative antifungal classes exist, for example allylamines and morpholines, but they can only be administered topically due to poor systemic efficacy [14]. The most common antifungal drug for systemic infections is amphotericin B, followed by triazoles such as fluconazole. The broad use of licensed antifungals and relative lack of alternatives has contributed to an increase in antifungal resistance within these species [12]. Currently licensed topical formulations encounter difficulties with delivering sufficient cidal concentrations of antifungals to the site of infection, resulting in the survival and spread of fungal populations more tolerant to drug therapy. This is due partly to the highly hydrophobic nature of antifungal molecules, which are limited by the quantity that can be loaded into a topical formulation base by physiochemical issues such as solubility [15].

Antimicrobial peptides have long been of interest to those involved in drug development as an alternative class of therapies to relieve the burden of antimicrobial resistance [16]. Existing within nature as mediators of the innate immune response, they are particularly promising given that they possess multiple microbial targets, thereby reducing the chance of resistance development. Microbial targets include fungal membranes and molecules involved within intracellular biomolecular processes such as protein replication and DNA synthesis [17]. Key to antimicrobial efficacy and selectivity is the hydrophobic: charge balance of the peptide motif, which ultimately defines membrane interactions and surfactant-like activity [18]. Several peptides selectively target fungal cells. Echinocandins, including caspofungin, micafungin and anidulafungin, are an existing class of licensed peptide antifungals that inhibit β-(1,3)-glucan synthase, responsible for the production of β-(1,3)-glucan essential for fungal cell wall integrity [19]. The cationic human salivary peptide histatin-5 is capable of producing reactive oxygen species and binds to fungal membrane receptors, entering cells to target mitochondria and resulting in fungal cell death [20,21].

**Figure 1:**
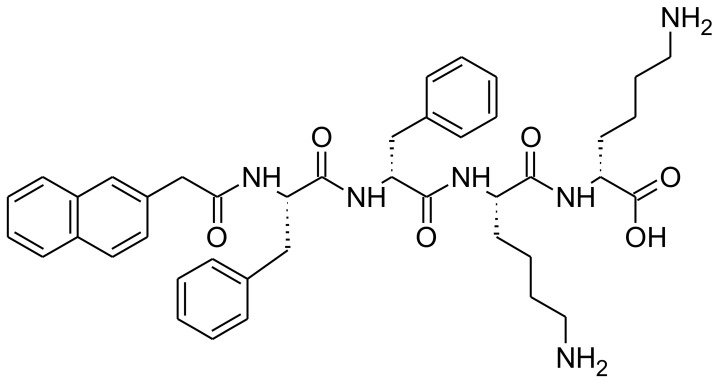
Chemical structure of NapFFKK-OH hydrogelator.

An interesting group of peptides have the ability to assemble into defined nanomaterial architectures, including nanofibers and nanotubes, in response to changes in physiological stimuli, including pH, ionic strength, temperature and the presence of specific enzymes [22]. Of particular promise to antimicrobial drug delivery is the ability of these peptides to assemble into supramolecular hydrogels composed of nanofibres. Our group has been successful in designing a variety of ultrashort peptide motifs (≤7 amino acids) that demonstrate inherent hydrogel forming capability and antimicrobial activity within a single molecular motif [23,24,25]. This overcomes the formulation issues associated with current topical therapies, whereby the quantity of drug loaded into a hydrogel base is restricted by drug solubility and disruption of the hydrogel’s mechanical properties. Antimicrobial peptide hydrogels have the potential for future application within the areas of anti-infective biomaterials and wound healing. However, peptide gels have not been studied for possible use within antifungal applications. This paper will outline the antifungal efficacy of our (naphthalene-2-ly)-acetyl-diphenylalanine-dilysine-OH (NapFFKK-OH) motif (Figure 1) as a peptide hydrogel therapy against a variety of fungal pathogens including *Aspergillus niger*, *Candida albicans*, *Candida glabrata*, *Candida dubliniensis* and *Candida parapsilosis*.

## 2. Results and Discussion

### 2.1. Hydrogel Formation and Characterisation

**Figure 2:**
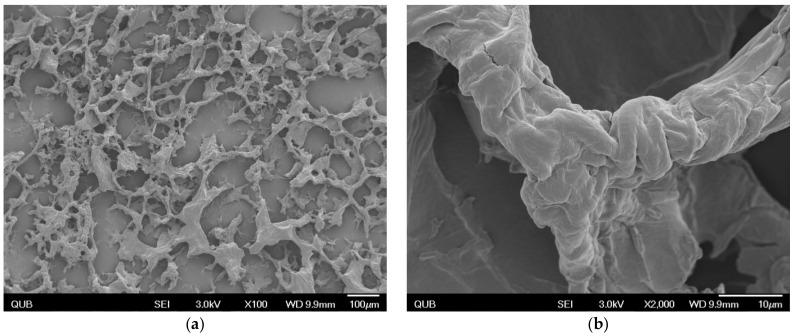
SEM images of 2% *w*/*v* NapFFKK-OH peptide at (**a**) 100× magnification, scale bar = 100 µm; (**b**) 2000× magnification, scale bar = 100 µm.

As previously demonstrated by our group, NapFFKK-OH was shown to form hydrogels at concentrations of 1% *w*/*v* and above using a formulation method of pH induction [23]. Scanning electron microscopy (SEM) images (Figure 2) confirmed NapFFKK-OH hydrogels were of nanofibrous architecture despite the presence of some drying artefacts due to flash freezing of the hydrogel formulation. These are formed by intermolecular interactions, primarily π–π and Van der Waal’s, between phenyl groups within the naphthalene and phenylalanine subunits. NapFFKK-OH hydrogels demonstrate random entanglements of densely packed three-dimensional fibres formed from β-sheet peptide secondary structures commonly observed for ultrashort peptide motifs based on the diphenylalanine (FF) sequence. Our group has previously characterized rheological properties of NapFFKK-OH, demonstrating its rigid mechanical properties typical of a self-supporting gel, with storage moduli (*G*′) observed to be larger than loss moduli (*G*″) by at least an order of magnitude (*G*′ = 200 Pa, *G*″ = 20 Pa, 2% *w*/*v*) [23,26].

### 2.2. Fungal Susceptibility

**Figure 3:**
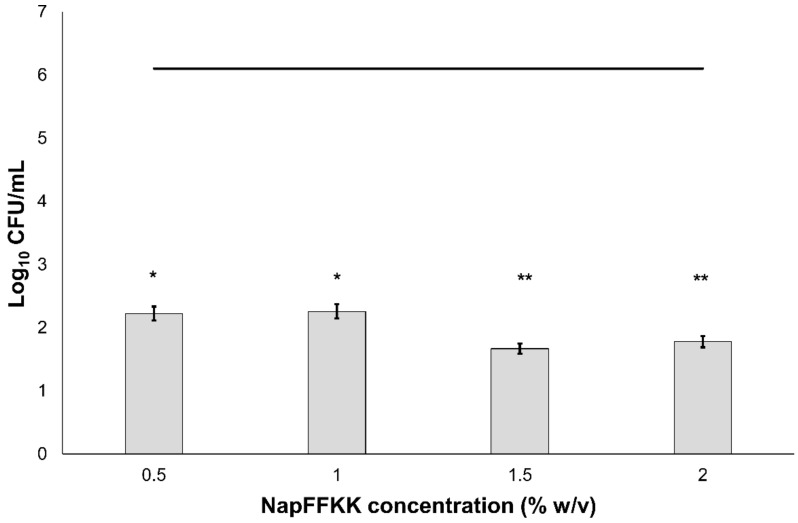
Fungal viability counts (Log_10_ CFU/mL) of *Aspergillus niger* CABI 017454 after 24 h exposure to NapFFKK-OH. Black line represents negative growth control (fungi only). *: *p* < 0.05, **: *p* < 0.01 significant difference between Log_10_ CFU/mL NapFFKK-OH treatment and the negative control.

**Figure 4:**
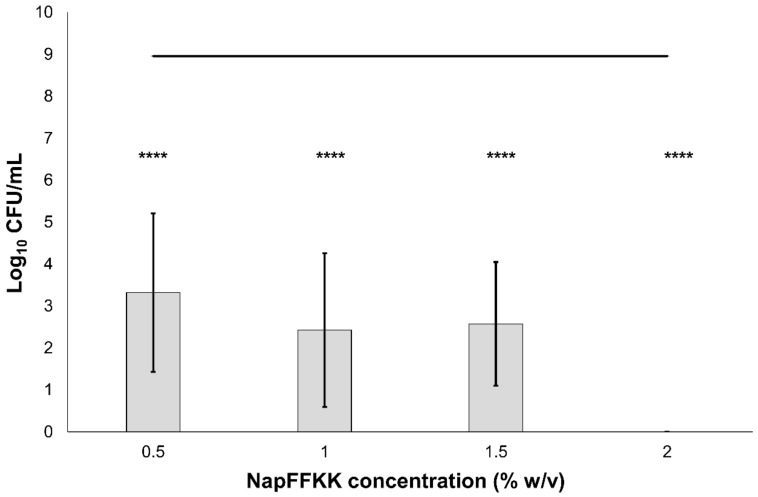
Fungal viability counts (Log_10_ CFU/mL) of *Candida albicans* NCTC 3179 after 24 h exposure to NapFFKK-OH. Black line represents negative growth control (fungi only). ****: *p* < 0.0001 significant difference between Log_10_ CFU/mL NapFFKK-OH treatment and the negative control.

**Figure 5:**
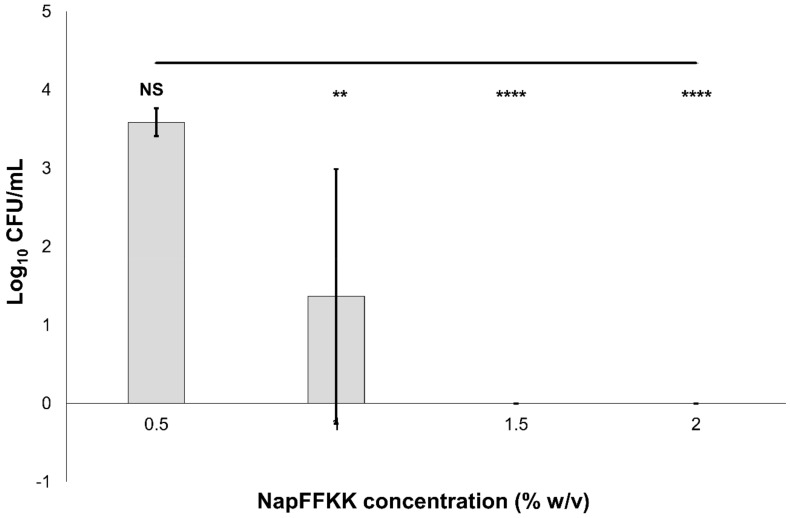
Fungal viability counts (Log_10_ CFU/mL) of *Candida parapsilosis* ATCC 22019 after 24 h exposure to NapFFKK-OH. Black line represents negative growth control (fungi only). NS: no significant difference (*p* ≥ 0.05), **: *p* < 0.01, ****: *p* < 0.0001 significant difference between Log_10_ CFU/mL NapFFKK-OH treatment and the negative control.

**Figure 6:**
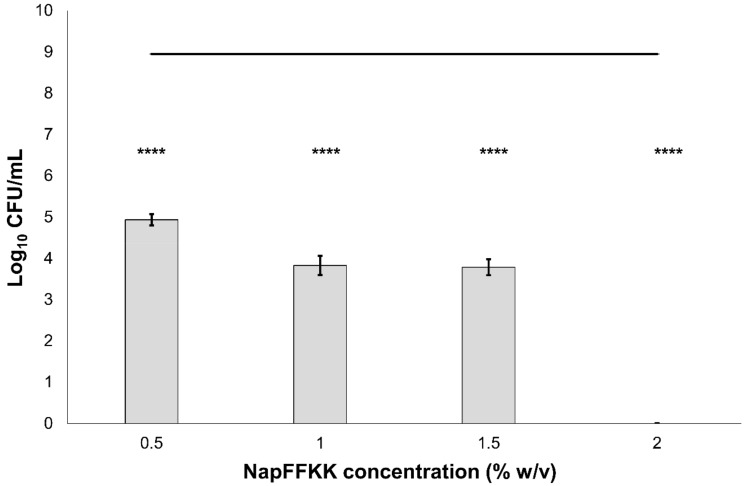
Fungal viability counts (Log_10_ CFU/mL) of *Candida dubliniensis* DC24 9 after 24 h exposure to NapFFKK-OH. Black line represents negative growth control (fungi only). ****: *p* < 0.0001 significant difference between Log_10_ CFU/mL NapFFKK-OH treatment and the negative control.

**Figure 7:**
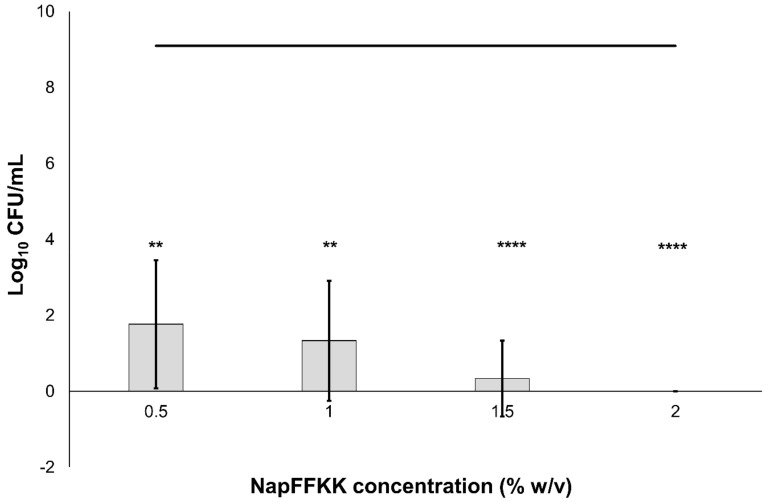
Fungal viability counts (Log_10_ CFU/mL) of *Candida glabrata* ATCC 2001 after 24 h exposure to NapFFKK-OH. Black line represents negative growth control (fungi only). **: *p* < 0.01, ****: *p* < 0.0001 significant difference between Log_10_ CFU/mL NapFFKK-OH treatment and the negative control.

The antifungal activity of NapFFKK-OH was assessed after incubation with a range of clinically relevant pathogens commonly presenting in immunocompromised patients with fungal infections. These included *Aspergillus niger* (Figure 3) and four separate species of *Candida (glabrata*, *albicans*, *parapsilosis* and *dubliniensis*) (Figure 4, Figure 5, Figure 6 and Figure 7, Figure S4 and Figure S5) from a mixture of reference strains (i.e., ATCC, NCTC, CABI, NCYC) and clinical isolates of *Candida dubliniensis* sampled from the oral cavity of diabetic (DC24 9) and non-diabetic (NDC19) patients. Significant reduction in fungal viability, measured as log_10_ colony forming units per mL (CFU/mL), was observed against each fungal isolate after 24 h incubation with NapFFKK-OH at concentrations corresponding to hydrogel formation (≥1% *w*/*v*). A 3 log_10_ CFU/mL reduction, commonly employed as a marker for antimicrobial efficacy due to its links with minimum inhibitory concentrations (99.9% reduction in viability) [27], was also observed at gelation concentrations and above. *Aspergillus niger* is a significant threat to immunocompromised patients due to its opportunistic nature and existence as spores throughout the environment. NapFFKK-OH was able to produce greater than 4 log_10_ CFU/mL reduction at concentrations of 0.5% *w*/*v* and above (Figure 3). *Aspergillus*’ role in food spoilage means NapFFKK-OH may have wider application within the food industry [28]. Future studies relating to NapFFKK-OH’s efficacy against *Aspergillus*’ spores and biofilm phenotype would provide useful insight into its wider application within the pharmaceutical and food industry. 

Although several intracellular targets have been identified for fungi, it is likely that NapFFKK-OH’s main mode of action is via disruption of fungal cell membranes given its broad-spectrum antimicrobial action [23]. Whilst fungal susceptibility assays demonstrate improved antifungal activity at gelation concentrations (≥1% *w*/*v*), this is likely to be concentration-dependent. Previous studies have shown a link between nanofibre formation and improved antimicrobial activity. The establishment of β-sheet secondary structures has been associated with an increase in peptide amphipathicity, which may enable specific targeting of anionic components within the fungal cell membrane [29]. Recent research has also hypothesised that hydrogel assembly, conformation, mechanical properties and molecular folding may be of importance in determining whether hydrogels possess antimicrobial selective activity via improved interactions with and suctioning of anionic cell membrane components [30,31,32]. As with the β-hairpin peptide hydrogels of the Schneider group [33], a combination of localised cationic charge throughout the porous network and hydrogel surface (Figure 2) is likely to be responsible for NapFFKK-OH’s broad spectrum antifungal and antimicrobial efficacy [23]

Similar to mammalian cells, an overall neutral charge is associated with fungal cell membranes due to the predominance of sterols (ergosterol in fungi and cholesterol in mammals). However, the presence of local pockets of anionic charge may lead to greater selectivity for fungal membranes compared to mammalian cells. The cationic charge of peptides is important to facilitate binding with anionic charged cell wall/membrane components, including mannoproteins present in yeasts such as *Candida* [29]. Fungi also possess a cell wall composed of mainly polysaccharides such as glucan, chitin, mannan and laminarin in *Candida*. Wang and colleagues recently demonstrated that the cationic peptide Polybia-MPI, isolated from wasp venom, could selectively bind to laminarin present on the cell wall surface of *Candida glabrata* and may also serve as a target for NapFFKK-OH, leading to membrane permeabilisation and/or disruption [34]. Previously observed insensitivity to cationic antimicrobial peptides, histatin-5 and magainin, by *Candida glabrata* was not observed for NapFFKK-OH at the concentrations employed in this study with greater than 7 log_10_ CFU/mL reduction against ATCC 2001 at concentrations of 0.5% *w*/*v* and above (Figure 7) and total kill against ATCC 90030 at 2% *w/v* (Figure S6) [35]. The interaction between antimicrobial peptides and fungal membranes is complex, especially given the eukaryotic similarities to mammalian membranes, and to date they have not been satisfactorily elucidated.

### 2.3. Mammalian Cell Cytotoxicity and Viability

**Figure 8:**
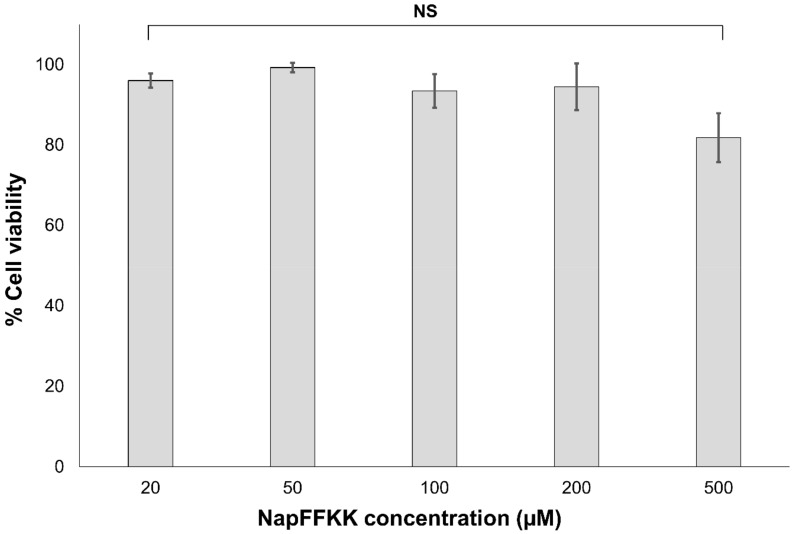
Percentage cell viability of NCTC clone 929 (ATCC CCL-1) cells after 24 h exposure to varying concentrations of NapFFKK-OH using CellTiter 96^®^ AQueous One Solution assay. NS: no significant (*p* ≥ 0.05) difference between NapFFKK-OH treatment and the negative control.

**Figure 9:**
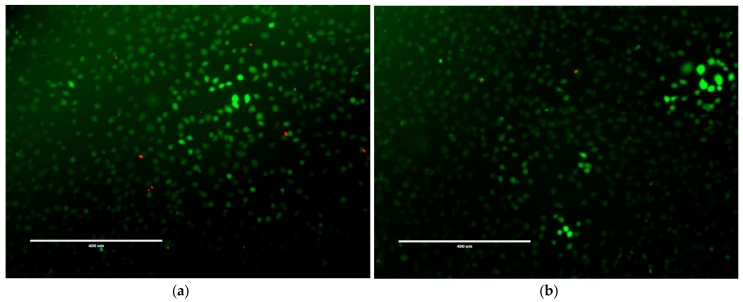

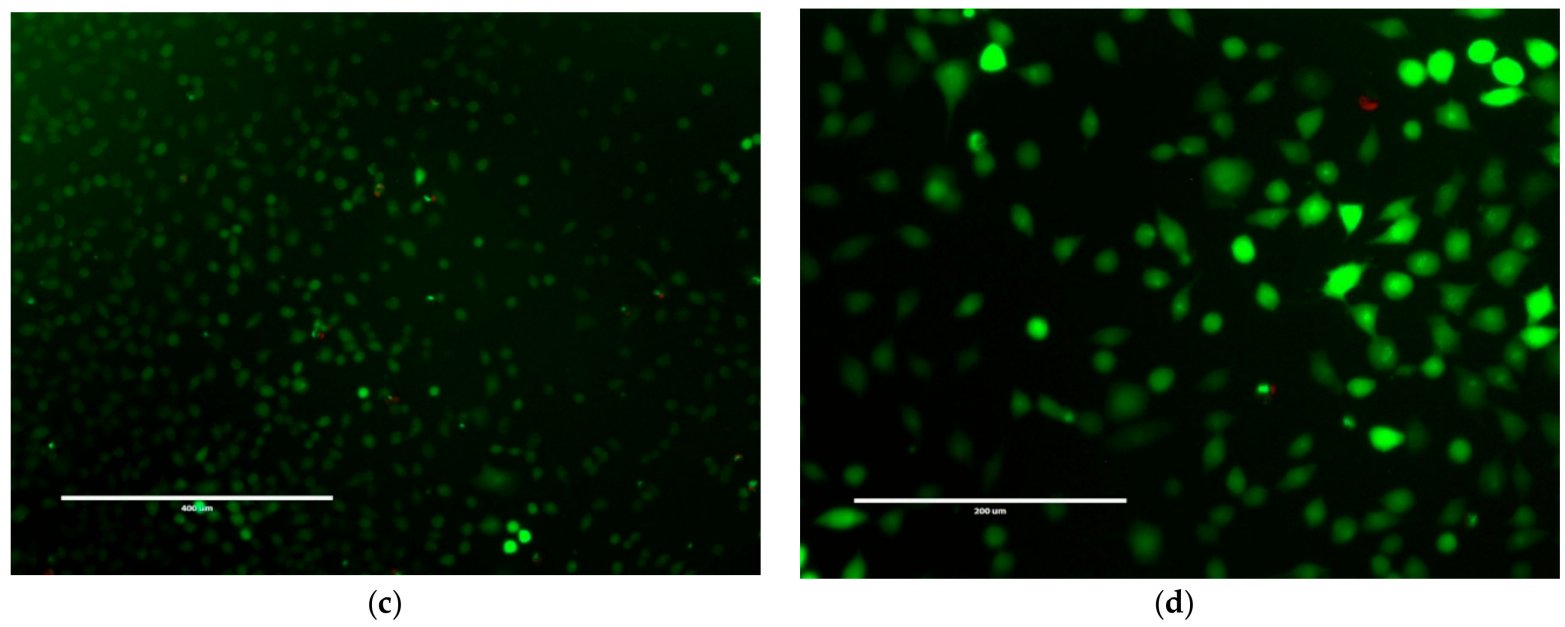
LIVE/DEAD^®^ fluorescence of NCTC 929 cells with (**a**) 20 µM; (**b**) 100 µM; (**c**) 200 µM; (**d**) 500 µM of NapFFKK-OH. Green staining indicates live cells, red staining indicates dead cells. Each image was taken after 24 h incubation with NapFFKK-OH, scale bar: (**a**–**c**) = 400 µm (**d**) = 200 µm.

Cell cytotoxicity and viability of NapFFKK-OH against NCTC 929 subcutaneous cells and ARPE-19 retinal epithelium cells were established using CellTiter 96^®^ AQueous One Solution (Promega, Southampton, UK) and LIVE/DEAD^®^ viability/cytotoxicity fluorescent (Thermo Fisher Scientific (Waltham, MA, USA) assays. NCTC 929 was employed as an International Organisation for Standardisation (ISO) cell line commonly utilised within biomaterial and medical device in vitro toxicity testing. ARPE-19 cells were used as a comparator ocular derived cell line, providing preliminary data for assessing NapFFKK-OH’s feasibility as a topical formulation for treating fungal ocular infections. Naphthalene was purposely chosen as an aromatic driver of hydrogelation within NapFFKK-OH due to a more established safety profile relative to similarly employed groups, for example the fluorenylmethyloxycarbonyl (Fmoc) moiety [25,36]. Concentrations tested were in the micromolar range (μM), representative of sustained breakdown of NapFFKK-OH at infected sites (e.g., due to enzymatic proteases) and subsequent exposure of cells to solubilised NapFFKK-OH. Previous work by our group demonstrated that NapFFKK-OH hydrogels possess over 80% cell viability (NCTC 929, 24 h) at 1% *w*/*v* [23]. Xu’s group established that NapFF has greater than 90% viability at 200 μM against HeLa cells [36]; however, it was important to establish whether cytotoxicity was affected by the increase in cationic character provided by the addition of two lysines to NapFF. No significant toxicity was demonstrated in the micromolar range (up to 1000 µM) against either NCTC 929 (Figure 8) or ARPE-19 cells (Figure S7) using MTS viability reagent after 24 h. However, after 48 h incubation with ARPE-19 cells, viability was reduced to its lowest observed value of 78.4% (1000 µM), with significant toxicity demonstrated for NapFFKK-OH at concentrations of 200 µM. This is just below ISO’s own 80% cell viability threshold value commonly accepted as biocompatible for in vitro studies. It is likely that prolonged 48 h exposure to cationic NapFFKK-OH results in disruption of mammalian membranes. No significant haemolysis was observed for NapFFKK-OH after exposure to equine erythrocytes, with a negligible value of <2% haemolysis observed at concentrations up to 500 µM (Figure S8). This serves as a useful indicator of the peptide’s ability to perturb mammalian cell membranes. A LIVE/DEAD^®^ assay employed alongside fluorescent microscopy confirmed NapFFKK-OH’s high cell compatibility after 24 h, evidenced by the propensity of green staining due to the conversion of calcein AM to calcein by viable NCTC 929 cells at concentrations up to 500 µM (Figure 9). Cell images obtained by optical microscopy (Figure S9) showed the presence at these concentrations of healthy NCTC 929 cells, which possessed arm-like projections associated with viable cells, enabling them to adhere to microtitre plates. Overall, these results suggest that NapFFKK-OH, like the majority of other antimicrobial peptide formulations currently within clinical trials [37], has the greatest promise as a topical therapy.

## 3. Conclusions

In conclusion, NapFFKK-OH represents an innovative formulation with the ability to form nanofibrous structures with inherent antifungal and hydrogel forming properties. Possessing such functionalities within a single molecular motif is important in order to overcome the formulation issues (drug loading, solubility) associated with current antifungal topical therapies. These limit the concentration of antifungal drug that can be delivered to infected sites, resulting in treatment failure and the survival/spread of exposed fungal isolates with increased resistance characteristics. NapFFKK-OH acts as a broad-spectrum antimicrobial, having previously been proven by our group to possess bactericidal action against planktonic and biofilms forms of Gram-positive and Gram-negative bacteria implicated in medical device infection [23]. Collectively, these observations suggest that NapFFK-OH antimicrobial activity is due to the targeting of cell membranes. Future work should focus on tailoring the primary peptide structure to selectively target only specific pathogenic fungal or bacterial species. It is increasingly becoming the view within modern medicine that broad-spectrum eradication of microbial flora is undesirable. Previous studies have suggested that modifying the lipophilic component of peptides with cholesterol and vitamin E results in compounds with unique antifungal activity [38]. This may be one avenue to explore if hydrogel-forming ability can be maintained. Similar to many other antimicrobial peptides, NapFFKK-OH may also prove to have synergistic benefits when combined with existing antifungal therapy, leading to reduced dosing, side effects and enhanced antifungal efficacy. The low molecular weight, ultrashort nature of NapFFKK-OH means it would be relatively cost-effective to upscale its manufacture to clinically relevant pharmaceutical production compared to larger-chain antimicrobial peptides isolated from biological sources. Significant challenges exist relating to the development of peptide therapies, especially as unfavourable biological stability and toxicity profiles limit their wider systemic administration. However, this work provides an important example of an ultrashort peptide hydrogelator that may contribute to the development of future antifungal and antimicrobial nanomaterial therapies for topical and biomaterial applications (wound dressings, medical devices). This is especially relevant given the increasing resistance to standardly employed antifungals and lack of alternative therapies available to healthcare professionals and patients.

## 4. Materials and Methods

### 4.1. Materials

Fmoc and Boc protected amino acids, *N*,*N*,*N*′,*N*′-tetramethyl-*O*-(1*H*-benzotriazol-1-yl)uronium hexafluorophosphate (HBTU), *N*,*N*-diisopropylethylamine (DIEA) 1-hydroxybenzotriazole (HOBt) and Wang resin (mesh size 100–200, 0.65 mmol/g) were obtained from Novabiochem, Merck KGaA (Darmstadt, Germany). LIVE/DEAD^®^ Viability/Cytotoxicity fluorescent assay was supplied by Thermo Fisher Scientific (Waltham, MA, USA). CellTiter 96^®^ AQueous One Solution, containing the cell viability reagent [3-(4,5-dimethylthiazol-2-yl)-5-(3-carboxymethoxyphenyl)-2-(4-sulfophenyl)-2H-tetrazolium (MTS), was purchased from Promega (Southampton, UK). 37% Hydrochloric acid (HCl), acetonitrile (HPLC grade, ≥99.93%), 2-naphthaleneacetic acid (>99% purity), Whatman pH indicator paper (pH 1–14), was obtained from Sigma-Aldrich (Gillingham, UK). Sterile Nunc 96-well microtitre plates and sodium hydroxide (NaOH) ≥99.0% pellets were purchased from VWR International (Lutterworth, UK). NCTC clone 929 (ATCC CCL-1) murine fibroblast subcutaneous connective tissue cells and ARPE-19 human retinal pigmented epithelium cells (ATCC CRL-2302) were sourced from LGC Standards (London, UK). Fresh defibrinated equine erythrocytes were purchased from Laboratory Supplies & Instruments Ltd. (Antrim, UK). Aspergillus niger CABI 017454, Candida glabrata ATCC 2001, Candida glabrata ATCC 90030, Candida albicans NCYC 610, Candida albicans NCTC 3179, Candida parapsilosis ATCC 22019, Candida dubliniensis NDC19 (isolated from the mouth of a non-diabetic patient) and Candida dubliniensis DC24 9 (isolated from the mouth of a diabetic patient) were kindly donated by Dr Fionnuala Lundy, School of Medicine, Dentistry and Biomedical Sciences, Queen’s University Belfast.

### 4.2. Methods

#### 4.2.1. Synthesis

NapFFKK-OH peptide was synthesised using standard Fmoc solid-phase peptide methods and using a nitrogen bubbler apparatus, as previously outlined by our group [23]. Wang resin preloaded with Fmoc–Lys(Boc)-OH,) (Novabiochem, Merck KGaA, Darmstadt, Germany) was utilized to produce carboxylic acid terminated NapFFKK-OH, upon cleavage with a mixture of 95% *v*/*v* trifluoracetic acid, 2.5% *v*/*v* triisopropylsilane and 2.5% *v*/*v* thioanisole for 3 h at room temperature. Standard HBTU coupling with DIEA and HOBt was performed in dimethylformamide (DMF) with 4-fold molar excess of DIEA and 3-fold excess of Fmoc-protected amino acid or 2-naphthaleneacetic acid used for coupling for 3 h at room temperature. Peptide precipitation was achieved in cold diethyl ether −20 °C). Crude product was dissolved in ethyl acetate and subjected to a series of washes with 1 mM HCl (3 × 50 mL) and water (3 × 50 mL) and dried over anhydrous magnesium sulphate (MgSO_4_). NapFFKK-OH identity was confirmed using electrospray mass spectroscopy (Figure S1) (Thermo Finnigan LCQ Deca ion trap, Thermo Fisher Scientific) and ^1^H NMR analysis (Figure S2) (Varian Unity Inova 400 spectrometer, Varian systems, Palo Alto, CA, USA) in d_6_-DMSO. NapFFKK-OH purity was determined by reverse-phase HPLC (Agilent 1260 series, Agilent Technologies Ltd., Cork, Ireland), using a Gemini C18 column (250 mm × 4.6 mm) with a flow rate of 1.5 mL min^−1^ and gradient of 2–60% acetonitrile (30 min) in 0.05% TFA–water. NapFFKK-OH was found to have a purity greater than 95% (Figure S3).

#### 4.2.2. Hydrogel Formulation

Homogenous NapFFKK-OH hydrogels were prepared using a method of pH-triggered induction. NapFFKK-OH peptide powder is dissolved at higher pH (~9) upon titration with NaOH due to ionisation of the carboxylic acid groups and hydrogel formation proceeds after subsequent titration to pH 7.4 and reattachment of H^+^ to the carboxylate anion (COO^−^) using HCl. The steps required to formulate of 2% *w*/*v* NapFFKK-OH hydrogels are outlined in Table 1. Changes in pH were monitored using Whatman pH paper. Minimum gelation concentration (% *w*/*v*) was defined as the lowest concentration of NapFFKK-OH that formed a self-supporting hydrogel, observed via a gel inversion assay after 24 h development. Previously reported values by our group of 1% *w*/*v* minimum gelation concentration for NapFFKK-OH were confirmed [23].

#### 4.2.3. Scanning Electron Microscopy

NapFFKK-OH hydrogels were formulated as outlined above (Table 1). 80 μL of NapFFKK-OH hydrogel was pipetted onto the SEM sample mount using a Gilson Microman E positive displacement pipette (Gilson, Birmingham, UK) and subjected to flash freezing in liquid nitrogen and freeze dried. A JEOL JSM 6500 F SEM (JEOL, Freising, Germany) was used for SEM imaging at 3 kV, with each sample pre-coated with an 8 nm layer of gold.

#### 4.2.4. Fungal Susceptibility Assay

The ability of NapFFKK-OH to reduce fungal viability was assessed using a colony count assay adapted from a method previously utilised by our group [24]. *Aspergillus niger* CABI 017454, *Candida glabrata* ATCC 2001, *Candida glabrata* ATCC 90030, *Candida albicans* NCYC 610, *Candida albicans* NCTC 3179, *Candida parapsilosis* ATCC 22019, *Candida dubliniensis* NDC19 and *Candida dubliniensis* DC24 9 were inoculated and allowed to grow for 18–24 h at 37 °C in Sabouraud dextrose broth) containing chloramphenicol 0.05 g/L. The optical density was adjusted to 0.15 at 530 nm to 2 × 10^6^ CFU/mL in Sabouraud dextrose broth to prepare a working suspension of fungi, and 100 μL of this was added to a 96-well microtiter plate and challenged with a range (0.5–2.0% *w*/*v*) of NapFFKK-OH concentrations formulated as in Table 1. One hundred microliters of each concentration of NapFFKK-OH was transferred to the microtiter plate using a Gilson Microman E positive displacement pipette. The positive control consisted of 100 μL of the working fungal suspension and 100 μL of PBS. The negative control consisted of 100 μL of sterile Sabouraud dextrose broth and 100 μL of pH 7.4 PBS. The challenge plates, containing NapFFKK-OH, were incubated for 24 h at 37 °C and viable counts obtained via Miles and Misra counting after plating 10 μL samples from the challenge plates on Sabouraud dextrose agar. Results were displayed as the mean (Log_10_ CFU/mL) of three replicates and tests were performed in triplicate.

#### 4.2.5. Cell Cytotoxicity and Viability Assays

Mammalian cell cytotoxicity was assessed using murine fibroblast subcutaneous connective tissue NCTC clone 929 (ATCC CCL-1) and ARPE-19 retinal pigmented epithelium cells (ATCC CRL-2302) cell lines. NCTC 929 cells were cultured in Eagle’s Minimum Essential Medium (MEM) containing phenol red with Earle’s Salts and L-glutamine, supplemented with 10% horse serum (Invitrogen, Paisley, UK). ARPE-19 cells were cultured in Dulbecco’s Modified Eagle Medium (DMEM) containing 10% foetal bovine serum. Cells were grown at 37 °C and 5% CO_2_ and subcultured at 80–90% confluency. Subculturing involved the removal of spent media, washing with sterile PBS, and detachment of cell monolayers with 0.05% trypsin/0.53 mM disodium ethylenediaminetetraacetate dihydrate solution. Cells were cultured until at least third passage and inoculated at 1 × 10^4^ cells per well in a 96-well microtitre plate and incubated for 24 h. The medium was then removed and the cells exposed to 100 μL of a range of NapFFKK-OH concentrations (20–500 µM) for 24 h. Control wells included media only (100% viability, negative control) and 70% ethanol treated cells (100% kill, positive control). A LIVE/DEAD^®^ viability/cytotoxicity fluorescent assay was used alongside fluorescence microscopy (EVOS FL microscope) to assess cell viability. Following a 24 h incubation with each NapFFKK-OH concentration, NCTC 929 cells were incubated for 20 min with a mixture of 4 mM ethidium homodimer-1 and 2 mM calcein AM in pH 7.4 PBS. Viable cells were stained green due to the conversion of calcein AM to calcein, whilst nonviable cells were stained red due to ethidium homodimer-1. Three randomly chosen areas were selected for analysis. Cell cytotoxicity was also analysed using a MTS containing CellTiter 96^®^ AQueous One Solution viability assay. Cells were cultured as outlined above and following 24 h exposure to NapFFKK-OH, cell viability was assessed using MTS and allowed to develop for 1 h. Absorption was measured at 570 nm using a Tecan Sunrise plate reader (Tecan UK Ltd., Reading, UK). Cell viability was calculated using Equation (1) and reported as the mean of six replicates:

% Cell viability = [(Absorbance_570nm_ peptide treated− Absorbance_570nm_ negative control)/(Absorbance_570nm_ positive control − Absorbance_570nm_ negative control) × 100]. (1)

#### 4.2.6. Haemolysis Assay

NapFFKK-OH was assessed spectrophotometrically for its ability to induce haemoglobin release from fresh equine erythrocytes according to the method previously utilized by our group [18]. Fresh defibrinated equine erythrocytes were washed three times with equal volumes of PBS. After centrifugation for 15 min at 900× *g*, erythrocytes were resuspended 4% *v*/*v* in PBS. Equal volumes (100 µL) of the erythrocyte suspension were added to each well of a 96-well microtitre plate. Erythrocytes were subsequently exposed to varying concentrations of peptide, incubated at 37 °C for 1 h and centrifuged at 1000× *g* for five minutes. Aliquots of the supernatant were transferred to a fresh 96-well microtiter plate, and haemoglobin release measured spectrophotometrically at 405 nm using a Tecan Sunrise plate reader. As a positive control (100% haemolysis), erythrocytes were treated with 10% Triton X-100, whilst PBS (0% haemolysis) acted as the negative control. Results for all concentrations are reported as the mean of six replicates. Percentage haemolysis was calculated using Equation (2) below:

% Haemolysis = [(Absorbance_405nm_ peptide − Absorbance_405nm_ negative PBS control)/(Absorbance_405nm_ 10% Triton X − Absorbance_405nm_ negative PBS control) × 100]. (2)

#### 4.2.7. Statistical Analysis

Statistical analyses were performed using Microsoft Excel 2013 (Microsoft, Redmond, WA, USA) and GraphPad Prism 6 (GraphPad Software, La Jolla, CA, USA). Standard deviations were obtained at each concentration of NapFFKK-OH based on nine replicates (three replicates performed in triplicate) for quantitative fungal viability assays and mean values obtained. For cell cytotoxicity assays standard deviations and mean values were also obtained from six replicates at each concentration. Statistical analyses were employed using a one-way Analysis of Variance (ANOVA) and a Tukey–Kramer multiple comparisons test used to identify individual differences between a reduction in fungal viability for NapFFKK-OH relative to the negative PBS control. Similarly ANOVA was also utilised for statistical analysis of cell cytotoxicity data by comparison of percentage viability (MTS) for the NapFFKK-OH employed to the medium-only negative control (100% viability). Haemolysis data were compared by the same statistical method with percentage haemolysis compared to the PBS, negative, non-haemolytic, control (0% haemolysis). ANOVAs were employed rather than parametric Kruskal–Wallis tests as the data were demonstrated to be normally distributed using the Kolmogorov and Smirnov method. In all cases a probability of *p* < 0.05 denoted significance.

## Figures and Tables

**Table 1:** Stepwise formulation of a self-assembling pH-triggered 2% *w*/*v* NapFFKK-OH (500 µL).

Formulation Step	Constituent	Quantity
1	NapFFKK-OH	10 mg pre-weighed
2	Deionised H_2_O	200 µL (in 50 µL aliquots)
3	1 M NaOH	50 µL (in 10 µL aliquots)
4	Deionised H_2_O	200 µL (in 50 µL aliquots)
5	0.5 M HCl	20 µL (in 10 µL aliquots)
6	Deionised H_2_O	To 500 µL

## Supplementary Materials

The following are available online at www.mdpi.com/2310-2861/4/2/48/s1, Figure S1: Mass spectra NapFFKK-OH, Figure S2: ^1^H NMR of NapFFKK-OH, Figure S3: HPLC trace NapFFKK-OH, Figure S4: Fungal viability counts (Log_10_ CFU/mL) of *Candida albicans* NCYC 610 after 24 h exposure to NapFFKK-OH, Figure S5: Fungal viability counts (Log_10_ CFU/mL) of *Candida dubliniensis* NDC19 after 24 h exposure to NapFFKK-OH, Figure S6: Fungal viability counts (Log_10_ CFU/mL) of *Candida glabrata* ATCC 90030 after 24 h exposure to NapFFKK-OH, Figure S7: Percentage cell viability of ARPE-19 retinal pigmented epithelium cells (ATCC CRL-2302) after 24 h exposure to varying concentrations of NapFFKK-OH, Figure S8: Percentage haemolysis of equine erythrocytes after 1 h exposure to varying concentrations of NapFFKK-OH, Figure S9: Optical images of NCTC 929 cells with NapFFKK-OH.
