# Supplementary material for: Ultrashort Self-Assembling Peptide Hydrogel for the Treatment of Fungal Infections
Source: Gels. 2018 May 22;4(2):48. doi: 10.3390/gels4020048 (PMC6209295; doi:10.3390/gels4020048)
Supplement: Supplementary file 1 [file gels-04-00048-s001.pdf]

Supplementary material for

Ultrashort self-assembling peptide hydrogel for the treatment of fungal infections

Alyaa Albadr, Sophie M Coulter, Simon L Porter, Raghu Raj Singh Thakur and Garry Laverty*

Biofunctional Nanomaterials Group, School of Pharmacy, Queen's University Belfast, Medical Biology Centre, 97 Lisburn Road, Belfast, N. Ireland, BT97BL.

Contents

Figure S1. Mass spectra NapFFKK-OH……………………………………………………S2

Figure S2. ^{1}H NMR of NapFFKK-OH………………………………………………………S2

Figure S3. HPLC trace NapFFKK-OH…………………………………………………………S3

Figure S4. *Candida albicans* NCYC 610 fungal viability counts…………………………....S3

Figure S5. *Candida dubliniensis* NDC19 fungal viability counts…………………………....S4

Figure S6. *Candida glabrata* ATCC 90030 fungal viability counts…………………………S5

Figure S7. ARPE-19 human retinal pigmented epithelium cells (ATCC CRL-2302) viability……………………………………………………………………………………....S6

Figure S8. Haemolysis……………………………………………………………………….S7

Figure S9. Optical images of cell morphology NCTC 929…………………………………..S8

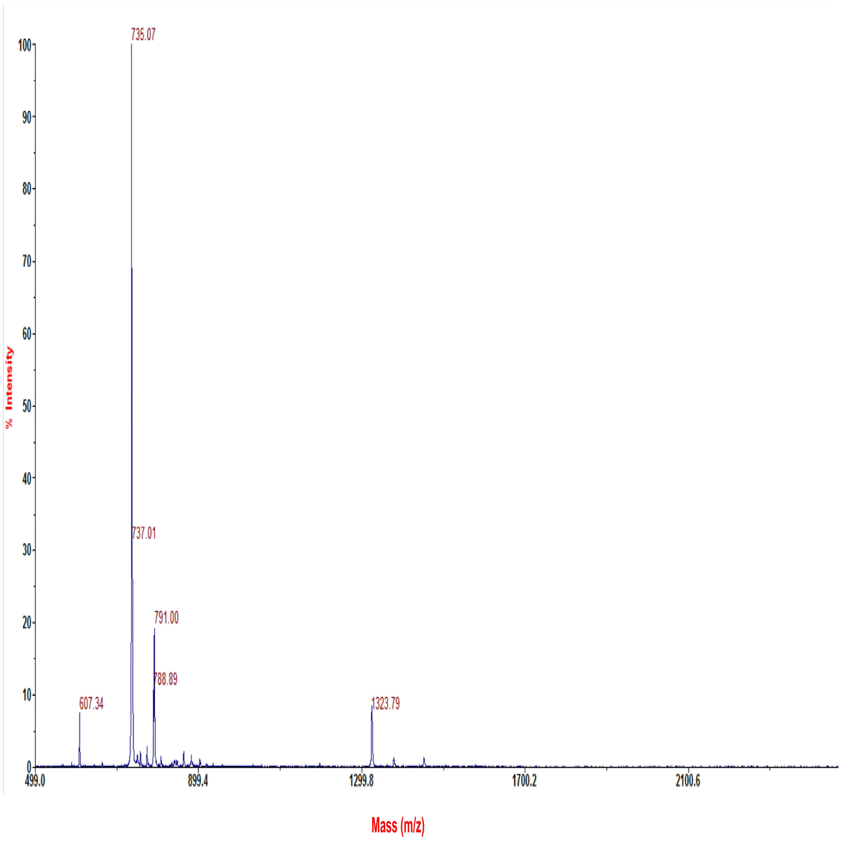

Figure S1. Mass spectra NapFFKK-OH, expected RMM 736.89 m/z.

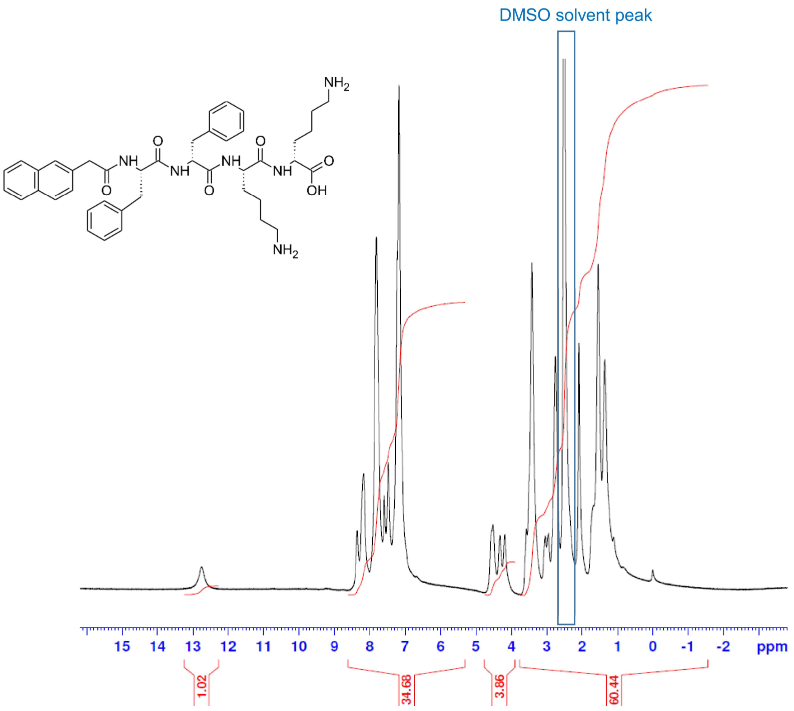

Figure S2. ^{1}H NMR, NapFFKK-OH, C_2D_6OS, TMS standard, 400 MHZ.

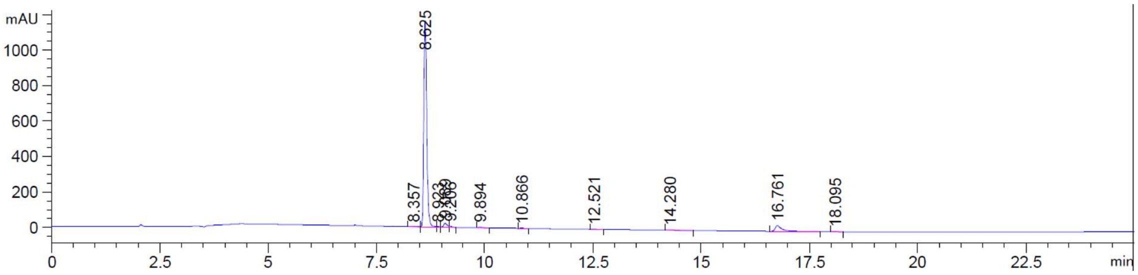

Figure S3. HPLC trace NapFFKK-OH, 95% purity.

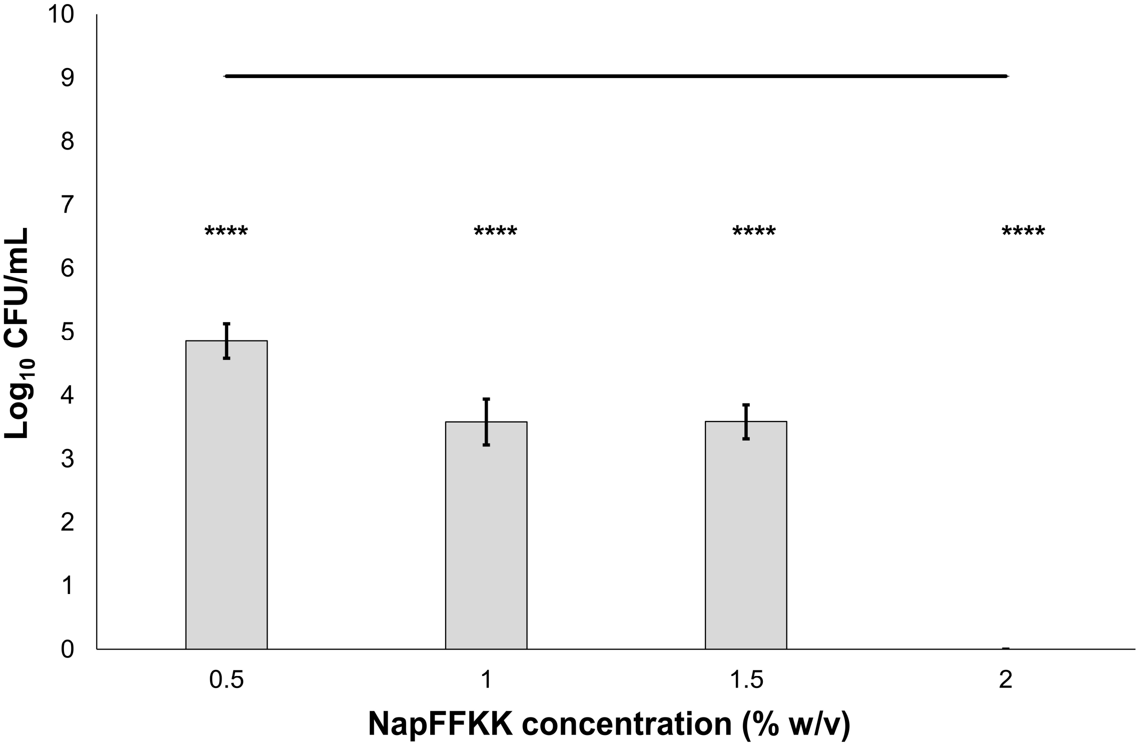

Figure S4. Fungal viability counts (Log_{10} CFU/mL) of *Candida albicans* NCYC 610 after 24 hours exposure to NapFFKK-OH. Black line represents negative growth control (fungi only). ****: $p<0.0001$ significant difference between Log_{10} CFU/mL NapFFKK-OH treatment and the negative control.

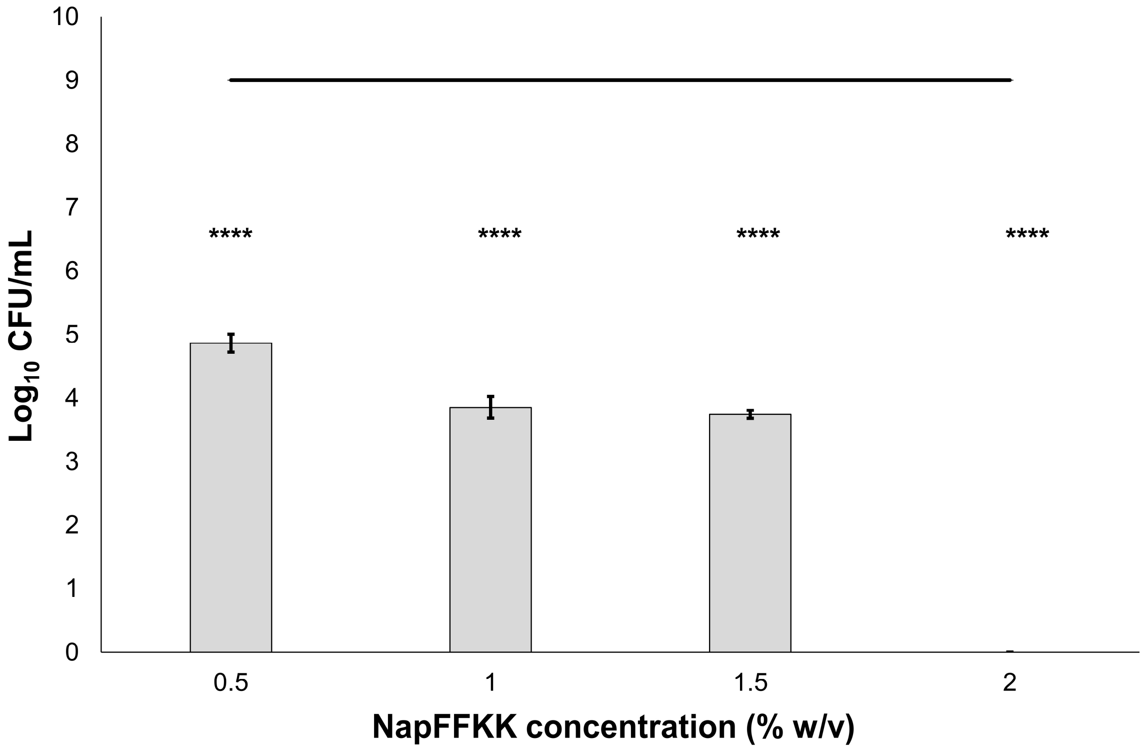

Figure S5. Fungal viability counts (Log_{10} CFU/mL) of *Candida dubliniensis* NDC19 after 24 hours exposure to NapFFKK-OH. Black line represents negative growth control (fungi only). ****: $p<0.0001$ significant difference between Log_{10} CFU/mL NapFFKK-OH treatment and the negative control.

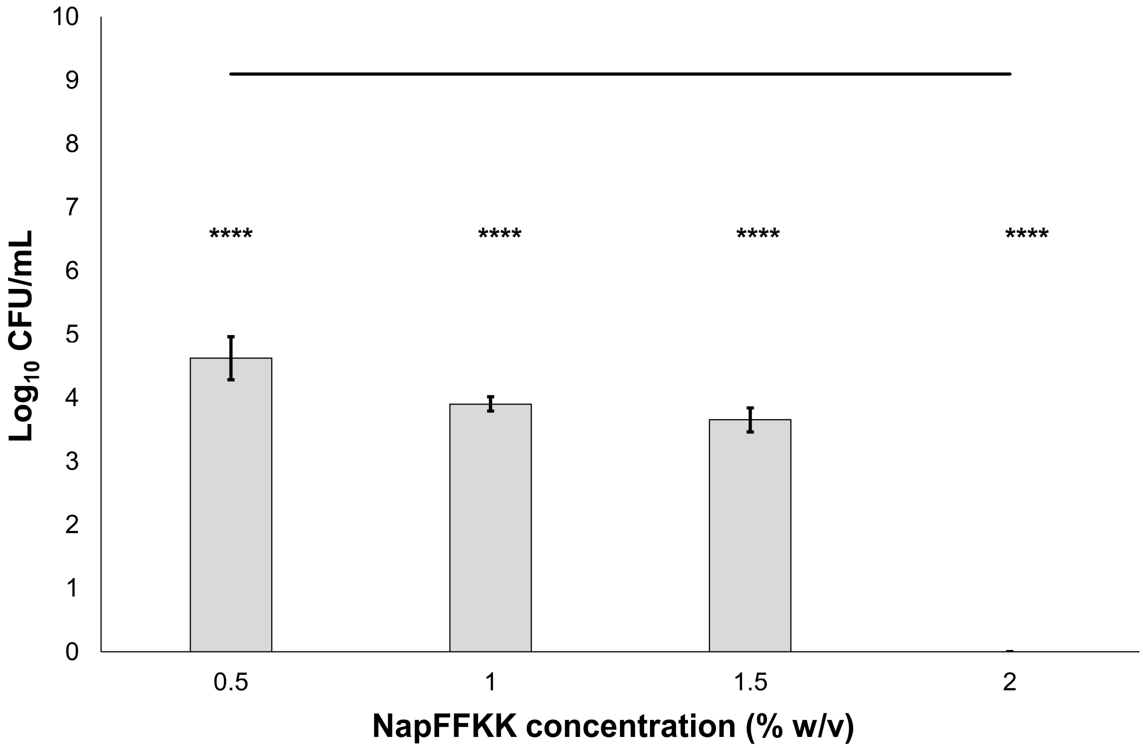

Figure S6. Fungal viability counts (Log_{10} CFU/mL) of *Candida glabrata* ATCC 90030 after 24 hours exposure to NapFFKK-OH. Black line represents negative growth control (fungi only). ****: $p<0.0001$ significant difference between Log_{10} CFU/mL NapFFKK-OH treatment and the negative control.

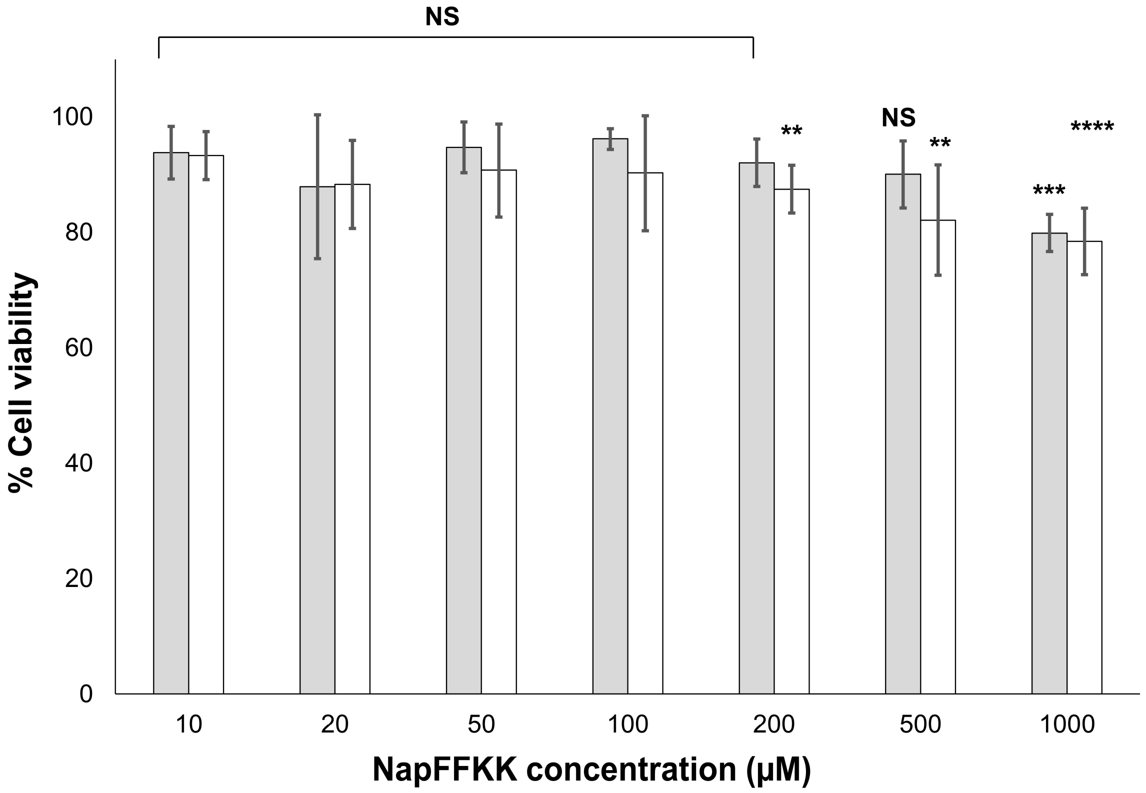

Figure S7. Percentage cell viability of ARPE-19 retinal pigmented epithelium cells (ATCC CRL-2302) after 24 hour (grey column) and 48 hour (white) exposure to varying concentrations of NapFFKK-OH. NS: no significant ($p \geq 0.05$), *: $p<0.05$, **: $p<0.01$, ***: $p<0.001$ ****: $p<0.0001$ difference between NapFFKK-OH treatment and the negative control.

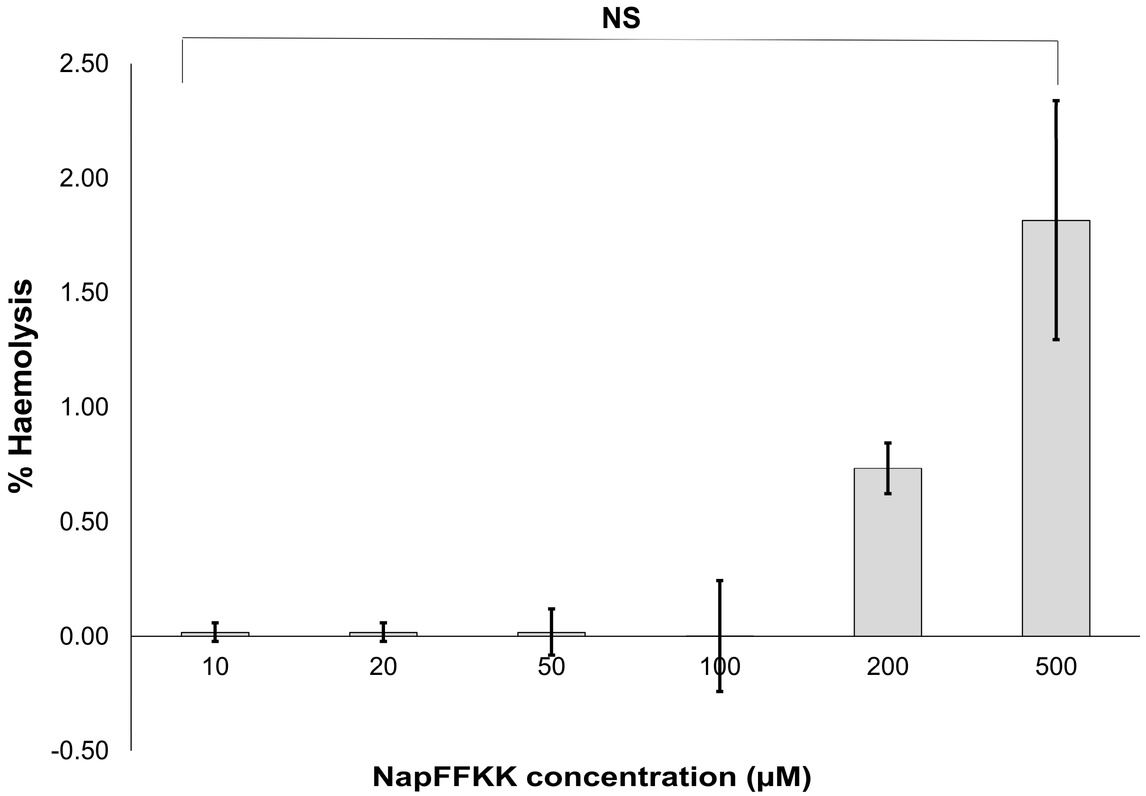

Figure S8. Percentage haemolysis of equine erythrocytes after 1 hour exposure to varying concentrations of NapFFKK-OH. Key: NS: no significant difference ($p \geq 0.05$) between the peptide nanotubes and the negative control (PBS).

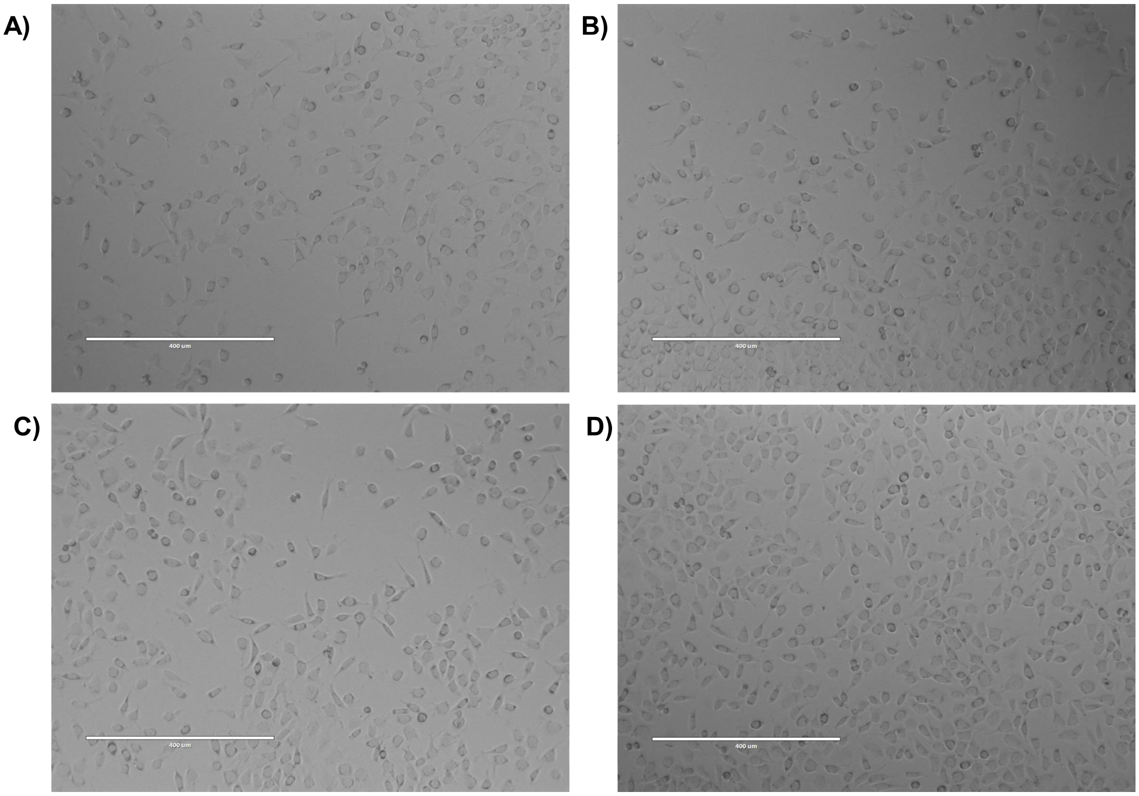

Figure S9. Optical images of NCTC 929 cells with (**A**) 20 μM; (**B**) 100 μM; (**C**) 200 μM; (**D**) 500 μM of NapFFKK-OH. Each image was taken after 24 hours incubation with NapFFKK-OH, scale bar = 400 μm.
